# Third Strain of Porcine Epidemic Diarrhea Virus, United States

**DOI:** 10.3201/eid2014.140908

**Published:** 2014-12

**Authors:** Douglas Marthaler, Laura Bruner, James Collins, Kurt Rossow

**Affiliations:** University of Minnesota Veterinary Diagnostic Laboratory, Saint Paul, Minnesota, USA (D. Marthaler, J. Collins, K. Rossow);; Swine Vet Center, Saint Peter, Minnesota, USA (L. Bruner)

**Keywords:** porcine epidemic diarrhea virus, complete genome analysis, phylogenetic analysis, PEDV strain, viruses, United States

**To the Editor**: In April 2013, porcine epidemic diarrhea virus (PEDV) was first reported (*1*). Since PEDV was detected, the virus has continued to spread throughout the United States and has now been reported in Mexico and Canada (*2*). PEDV was first reported in Europe in the 1970s and was later reported in Asia during the 1980s (*3*,*4*). PEDV outbreaks in Asia were more acute and severe than the PEDV outbreaks in Europe (*4*). In 2010, an increase, up to 100%, in illness and deaths in piglets was reported in China associated with PEDV infection (*4*–*6*). The original North American PEDV strains identified in 2013 caused severe illness and deaths in piglets and had a 99.5% nucleotide identity with Chinese strain AH2012 (*1*,*7*,*8*).

Recently, a North American PEDV variant-INDEL strain was identified, which has been previously described (*9*). This spike gene deletion has been described in global PEDV strains, which may correlate with a less severe clinical presentation of PEDV. Although the PEDV variant-INDEL strain (OH851) was first reported in February 2014 by the Ohio Department of Agriculture, the PEDV variant-INDEL strains were first detected in June 2013, which suggests that the original PEDV strain mutated or that 2 different PEDV strains were introduced concurrently into the United States (*2*).

The University of Minnesota Veterinary Diagnostic Laboratory has tested clinical samples from thousands of case-patients to determine the presence of PEDV by real-time reverse transcription PCR. Some of the PEDV-positive samples from case-patients were selected for PEDV spike gene sequencing per veterinarian’s request, whereas other samples were selected for complete genome sequencing to fulfill a grant objective. When PEDV was first detected in the United States, the University of Minnesota Veterinary Diagnostic Laboratory was only sequencing the PEDV spike gene segment to clarify the phylogenetic relationship between PEDV strains.

In February 2014, after the identification and analysis of PEDV variant-INDEL strains, we decided to sequence the complete PEDV genome by using next generation sequencing to clarify the phylogenetic relationship of the US PEDV strains (*7*). In January 2014, intestines from a deceased neonatal piglet, which had severe diarrhea, were positive for PEDV by real-time reverse transcription PCR, and PEDV was sequenced per request of the veterinarian. The sample was processed for next generation sequencing and analyzed by mapping to reference strain USA/Colorado/2013 (*7*). During the assembly of PEDV strain USA/Minnesota188/2014 (GenBank accession no. KM077139), a region of the spike only had 5× coverage and ambiguity bases in the consensus contig whereas the coverage across the remaining PEDV genome was 50×. The consensus contig was separated at this region, and the 2 separate contigs were created. After remapping, the 2 contigs were aligned back into a new consensus contig, and the new consensus contig was remapped to verify the accuracy of the contig, which had 50× coverage.

The PEDV strain Minnesota188 was aligned with the other complete genome of PEDV available in GenBank (n = 113); it had a 99.9% nucleotide percentage identity to Colorado/2013 and clustered in the North American clade II (Figure, panel A). The spike gene segment had a 99.7% nucleotide percentage identity (99.4% amino acid identity) with Colorado/2013 and clustered with the non–North American INDEL strains (Figure, panel B). The alignment indicated a spike gene nucleotide deletion at positions 164–169 (TTGGTG), which corresponded to amino acid deletion at positions 55 and 56. The spike gene amino acid alignment identified substitution at positions 23 (I), 31 (H), 57 (K), and 59 (E), which have not been identified in the complete PEDV genomes available from North America.

**Figure:**
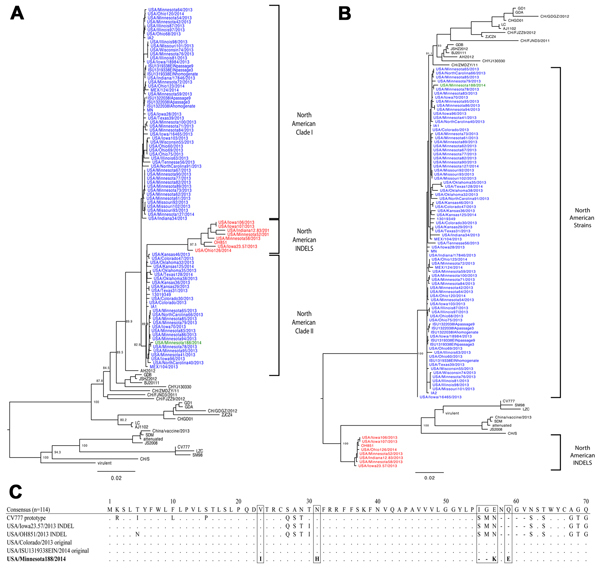
Complete genome (A) and spike gene (B) phylogenetic analysis using the maximum likelihood method with the general time reversible (GTR) nucleotide substitution model. Green indicates Minnesota188 strain, red indicates North American INDEL strains, and blue indicates the remaining North American porcine epidemic diarrhea virus (PEDV) strains. Scale bar indicates percentage of dissimilarity between sequences. C) Spike gene alignment of first 70 aa). The consensus represents 114 sequences used in the analysis. PEDV prototype strain CV777, 2 American original and INDEL PEDV strains are illustrated. Dots indicate amino acid residues matching the consensus are represented by dots; dashes represent amino acid deletions.

Thus far, 3 naturally occurring US PEDV strains have been identified: the original PEDV, the PEDV with changes in the spike gene (INDEL), and the PEDV strain (S2aa-del) reported in this article. The role of genetic changes in the US PEDV strains to clinical disease has yet to be reported. The clinical presentation of diarrhea in this case was reported as equally or more severe than such presentation in cases caused by the prototype PEDV Colorado/2013. Other factors such as concurrent infections and the rate of group exposure, which is rapid in most PEDV cases affecting neonatal piglets, may influence the clinical presentation.

Documenting PEDV variation is vital to understanding the natural evolution of the virus and possibly identifying portions of the genome associated with different clinical disease features. Animal studies are required to define the effects of these mutations on clinical disease, pathogenesis, immunity; these studies will be conducted in the future with the S2aa-del strain. A consistent model to properly evaluate these differences is required to control PEDV infection. The most compelling need is to understand how exposure by sows to different PEDV strains correlates with protection of piglets from clinical disease. Whether the PEDV S2aa-del strain will circulate in the North American swine population is not known.

## References

[1] Stevenson GW, Hoang H, Schwartz KJ, Burrough ER, Sun D, Madson D, Emergence of porcine epidemic diarrhea virus in the United States: clinical signs, lesions, and viral genomic sequences. J Vet Diagn Invest. 2013;25:649–54. 10.1177/1040638713501675 23963154

[2] Vlasova AN, Marthaler D, Wang Q, Culhane MR, Rossow KD, Rovira A, Distinct characteristics and complex evolution of PEDV, North America, May 2013–February 2014. Emerg Infect Dis. 2014;20:1620–8. 25279722 10.3201/eid2010.140491 PMC4193278

[3] Pensaert MB, de Bouck P. A new coronavirus-like particle associated with diarrhea in swine. Arch Virol. 1978;58:243–7. 10.1007/BF01317606 83132 PMC7086830

[4] Song D, Park B. Porcine epidemic diarrhoea virus: a comprehensive review of molecular epidemiology, diagnosis, and vaccines. Virus Genes. 2012;44:167–75. 10.1007/s11262-012-0713-1 22270324 PMC7089188

[5] Luo Y, Zhang J, Deng X, Ye Y, Liao M, Fan H. Complete genome sequence of a highly prevalent isolate of porcine epidemic diarrhea virus in South China. J Virol. 2012;86:9551. 10.1128/JVI.01455-12 22879620 PMC3416124

[6] Fan H, Zhang J, Ye Y, Tong T, Xie K, Liao M. Complete genome sequence of a novel porcine epidemic diarrhea virus in south China. J Virol. 2012;86:10248–9. 10.1128/JVI.01589-12 22923806 PMC3446595

[7] Marthaler D, Jiang Y, Otterson T, Goyal S, Rossow K, Collins J. Complete genome sequence of porcine epidemic diarrhea virus strain USA/Colorado/2013 from the United States. Genome Announc. 2013;1:. 10.1128/genomeA.00555-13 PMC3738886 23929470

[8] Jung K, Wang Q, Scheuer KA, Lu Z, Zhang Y, Saif LJ. Pathology of US porcine epidemic diarrhea virus strain PC21A in gnotobiotic pigs. Emerg Infect Dis. 2014;20:662–5 . 10.3201/eid2004.131685 24795932 PMC3966387

[9] Wang L, Byrum B, Zhang Y. New variant of porcine epidemic diarrhea virus, united states, 2014. Emerg Infect Dis. 2014;20:917–9. 10.3201/eid2005.140195 24750580 PMC4012824

